# AS03- and MF59-Adjuvanted Influenza Vaccines in Children

**DOI:** 10.3389/fimmu.2017.01760

**Published:** 2017-12-13

**Authors:** Amanda L. Wilkins, Dmitri Kazmin, Giorgio Napolitani, Elizabeth A. Clutterbuck, Bali Pulendran, Claire-Anne Siegrist, Andrew J. Pollard

**Affiliations:** ^1^The Royal Children’s Hospital Melbourne, Melbourne, VIC, Australia; ^2^Emory Vaccine Center, Emory University, Atlanta, GA, United States; ^3^Medical Research Council (MRC), Human Immunology Unit, University of Oxford, Oxford, United Kingdom; ^4^Oxford Vaccine Group, Department of Paediatrics, University of Oxford, The NIHR Oxford Biomedical Research Centre, Oxford, United Kingdom; ^5^Department of Pathology, Emory University School of Medicine, Atlanta, GA, United States; ^6^Department of Pathology, and Microbiology & Immunology, Stanford University, Stanford, CA, United States; ^7^Institute for Immunology, Transplantation and Infection, Stanford University, Stanford, CA, United States; ^8^University of Geneva, Geneva, Switzerland

**Keywords:** adjuvant, influenza vaccine, MF59, AS03, children

## Abstract

Influenza is a major cause of respiratory disease leading to hospitalization in young children. However, seasonal trivalent influenza vaccines (TIVs) have been shown to be ineffective and poorly immunogenic in this population. The development of live-attenuated influenza vaccines and adjuvanted vaccines are important advances in the prevention of influenza in young children. The oil-in-water emulsions MF59 and adjuvant systems 03 (AS03) have been used as adjuvants in both seasonal adjuvanted trivalent influenza vaccines (ATIVs) and pandemic monovalent influenza vaccines. Compared with non-adjuvanted vaccine responses, these vaccines induce a more robust and persistent antibody response for both homologous and heterologous influenza strains in infants and young children. Evidence of a significant improvement in vaccine efficacy with these adjuvanted vaccines resulted in the use of the monovalent (A/H1N1) AS03-adjuvanted vaccine in children in the 2009 influenza pandemic and the licensure of the seasonal MF59 ATIV for children aged 6 months to 2 years in Canada. The mechanism of action of MF59 and AS03 remains unclear. Adjuvants such as MF59 induce proinflammatory cytokines and chemokines, including CXCL10, but independently of type-1 interferon. This proinflammatory response is associated with improved recruitment, activation and maturation of antigen presenting cells at the injection site. In young children MF59 ATIV produced more homogenous and robust transcriptional responses, more similar to adult-like patterns, than did TIV. Early gene signatures characteristic of the innate immune response, which correlated with antibody titers were also identified. Differences were detected when comparing child and adult responses including opposite trends in gene set enrichment at day 3 postvaccination and, unlike adult data, a lack of correlation between magnitude of plasmablast response at day 7 and antibody titers at day 28 in children. These insights show the utility of novel approaches in understanding new adjuvants and their importance for developing improved influenza vaccines for children.

## Influenza and Non-Adjuvanted Vaccines

Influenza causes significant morbidity and mortality worldwide and it is estimated that 20–30% of children become infected with influenza each year (1). Although influenza infection often results in a self-limiting illness, young children are at increased risk of secondary pneumonia, hospitalization, and death (2, 3). The global incidence of influenza-associated acute lower respiratory infections (ALRI) in children less than 5 years old has been estimated at 20 million in 2008 (13% of all cases of pediatric ALRI) (4). In the same year, an estimated 28,000–111,500 deaths in children less than 5 years old were attributable to influenza-associated ALRI. The mortality burden is seen most in developing countries, where 99% of these deaths occurred. Moreover, influenza-related illness is responsible for substantial economic burden, contributing to an increasing number of outpatient appointments, missed school and antibiotic use in children (5, 6). Laboratory-confirmed influenza-related medical attendances in children less than 5 years of age have been reported as high as 27 emergency department visits per 1,000 children and 95 outpatient visits per 1,000 children (7).

Prevention of influenza is most effectively provided through vaccination and would ideally offer cross protection against drifted non-vaccine influenza virus strains. Children play an important role in transmission of influenza virus therefore the vaccination of this population is not only an important prevention strategy for direct protection but also indirect protection for the wider population (6, 8, 9). Licensed non-adjuvanted influenza vaccines for children include split or subunit inactivated influenza vaccines (IIV) and the live-attenuated influenza vaccine (LAIV). Young children are often naive to the influenza virus, have not previously been vaccinated, and are therefore unprimed. For this reason it is recommended that children receive two doses, 28 days apart, of an influenza vaccine in the first influenza season they receive immunization. Both IIV and LAIV have significant limitations for use in the pediatric age group. IIV is not licensed for use before the age of 6 months; it is poorly immunogenic in younger children, with an efficacy of 59% against confirmed influenza infection and 36% effectiveness against influenza-like illness (ILI) in children 6 months to 2 years old (10). Additionally, IIV provide poor cross-protection for mismatched influenza virus strains (10). LAIV has significantly better efficacy than IIV, with 55% fewer cases of confirmed influenza following LAIV compared with IIV (11). However, LAIV is not recommended for children less than 2 years of age due to increased rates of wheezing episodes postvaccination (11).

Currently, trivalent or quadrivalent influenza vaccination is recommended only for high-risk children in most countries—or for all children aged 6 months and older in certain developed countries including the US, UK, Australia, and Canada (12–15). In the UK the LAIV is funded through the routine immunization schedule for children aged 2–11 years (though roll out of the program to all these age groups is not yet complete) with evidence from surveillance data demonstrating direct protection in children against influenza infection and hospitalization (13, 16, 17). Conversely, LAIV has shown poor effectiveness in children in the US over the last three influenza seasons and was not recommended for the 2016–2017 season (18). Irrespective of these recommendations, uptake is suboptimal. Recent surveillance in the USA estimates only 26% of laboratory-confirmed influenza-associated pediatric deaths in children 6 months to 17 years having received an influenza vaccine prior to their illness (19). The common perception that influenza is a benign illness compared with other childhood infections, and the partial efficacy of influenza vaccines in young children limit its recommendations, its promotion and thus its uptake.

The limitations of IIV and LAIV in young children and the poor vaccination coverage result in one of the highest risk groups for influenza-related comorbidities receiving inadequate prevention and subsequent lack of herd protection for the remaining population. An approach to improving protection in children is the addition of adjuvants to the traditional IIV. Adjuvants are designed to enhance the immunological response to a vaccine and, when used for influenza vaccines, have afforded antigen dose sparing and improved cross-protection against non-vaccine influenza virus strains. A range of adjuvant formulations have been developed and there has been progress toward fully understanding the mechanisms involved their action in recent years. Historically there have been challenges involving the use of adjuvanted influenza vaccines in humans due to unacceptable adverse events (20–22). New and improved adjuvant systems have overcome this issue and there have been a number of approved adjuvanted influenza vaccines for children, including prepandemic, pandemic, and seasonal vaccines. This article provides an overview of the oil-in-water-adjuvanted influenza vaccines in children, MF59 and adjuvant systems 03 (AS03) (Table 1), highlighting their ability to provide improved protection for children against influenza.

**Table 1:** AS03- and MF59-adjuvanted vaccines for children.

	Adjuvant	Vaccines	Trade name	Hemagglutinin (HA) dose	Influenza vaccine type	Culture medium	Dose (pediatric)	Countries licensed for children
AS03	Oil-in-water emulsion Squalane, polysorbate 80 and α-tocopherol	A/H1N1 pandemic influenza vaccine	Pandemrix^®^ (GSK)	3.75 μg in 0.5 ml	Inactivated, split-influenza	Egg	0.25 ml	Europe

			Arepanrix^®^ (GSK)	3.75 μg in 0.5 ml	Inactivated, split-influenza	Egg	0.25 ml	Canada and Latin America

MF59	Oil-in-water emulsion Squalene, Tween 80 and Span 85	Seasonal trivalent influenza vaccine	Fluad^®^ (Novartis)	15 μg (in 0.5 ml) of each influenza strain surface antigen	Inactivated, subunit	Egg	0.25 ml	Canada
			Fluad Pediatric™ (Novartis)	7.5 μg (in 0.25 ml) of each influenza strain surface antigen				

		A/H1N1 pandemic influenza vaccine	Focetria^®^ (Novartis)	7.5 μg in 0.5 ml	Inactivated, subunit	Egg	0.5 ml	Europe and Latin America

			Celtura^®^ (Novartis)	3.75 μg in 0.5 ml	Inactivated, subunit	Madine-Darby canine kidney (MDCK) cells	0.25 ml	Some countries in Europe and Latin America

*GSK, GlaxoSmithKline*.

## Adjuvanted Influenza Vaccines

### MF59-Adjuvanted Influenza Vaccines

MF59 is an oil-in-water emulsion composed of squalene and two surfactants, Tween 80 and Span 85. Squalene is a naturally occurring oil synthesized in the human liver and is a direct precursor to cholesterol (23). The Chiron Vaccines company developed MF59 and it was first licensed as part of a seasonal influenza vaccine for the elderly population in Italy in 1997. Over 100 million MF59-containing vaccines have been distributed in over 30 countries around the world. The MF59-adjuavanted inactive trivalent influenza vaccine (TIV) [Fluad^®^, MF59-adjuvanted trivalent influenza vaccine (ATIV), Novartis Vaccines] contains 15 μg of each influenza strain surface antigen and the MF59 adjuvant and is administered as a 0.5 ml dose. It is licensed for adults aged 65 years and over. Fluad Pediatric^®^, a 0.25 ml dose, has now been licensed for children aged 6 months to 2 years in Canada since 2015. Two MF59-adjuvanted monovalent A/H1N1 pandemic influenza vaccines (Focetria^®^ and Celtura^®^, Novartis Vaccines) were licensed for children during the H1N1 influenza pandemic in 2009. Focetria^®^ is an egg-based inactivated subunit vaccine and Celtura^®^ a cell-culture-based inactivated subunit vaccine.

### AS03-Adjuvanted Influenza Vaccines

The AS03 adjuvant is an oil-in-water emulsion composed of squalene, polysorbate 80 and α-tocopherol (vitamin E). AS03 was first used in the prepandemic H5N1 vaccine Prepandrix (GlaxoSmithKline Biologicals s.a.) and was subsequently included in two influenza A(H1N1)pdm09 pandemic vaccines—Pandemrix^®^, GlaxoSmithKline Biologicals s.a., and Arepanrix^®^, GlaxoSmithKline Inc. Two AS03 formulations with differing amounts of tocopherol, AS03A (11.86 mg tocopherol) and AS03B (5.93 mg tocopherol), were used in the full dose and half dose vaccines, respectively. The 2009 A(H1N1) influenza pandemic was the first time the global deployment of a pandemic influenza vaccine had been undertaken. The benefit of using the AS03 adjuvant as part of a pandemic vaccine is its ability to induce high antibody titers with a reduced antigen dose (3.75 or 7.5 μg per strain compared with 15ug per strain in conventional TIV), making it possible to meet the global demand. Pandemrix^®^ was accepted for fast track authorization and had been given to less than 200 children aged 3–9 years before it was licensed (24). Approximately 4.7 million doses of AS03-adjuvanted A(H1N1) vaccines have been administered to children since 2009 (25).

## Immunology

### Effect of Adjuvants on Systemic Antibody Responses to Influenza Hemagglutinin (HA)

A number of clinical trials have demonstrated that the seroprotection induced by the MF59-adjuvanted vaccines is superior to TIV, even in the very young (26–33) or the elderly (34). The threshold of protection was defined in immunized adults as HAI titers ≥40 (50% protection from reinfection) or a fourfold rise from baseline (35). However, this was proven to be insufficient to protect infants and young children and new protective thresholds were defined by Black et al. (36). Here, children aged 16–72 months received two doses of an MF59 ATIV (Fluad^®^) or a TIV vaccine (Influsplit^®^). Follow up for any influenza like illness was confirmed by RT-PCR. Immunogenicity and surveillance data collected allowed the investigators to model a protective HAI titer that would give 80% (≥330) and 90% (≥629) protection in this age group. In a subsequent, similar study of children aged 14–24 months, 100% of children achieved thresholds of both ≥330 and ≥629 after two doses of MF59 ATIV in response to A/H1N1 and A/H3N2 vaccination in comparison with those receiving TIV (Imuvac^®^) where 8 and 47% of children achieved ≥330 to H1N1 and H3N2, respectively (29).

It seems that in all age groups, primed individuals respond more robustly to both TIV and ATIV vaccines (37–39).

### Adjuvants Induce Recruitment of Innate B Cells and IgM Production

Murine models have shown that influenza viruses cause inflammation of epithelial cells in the respiratory tract (40, 41). These innate inflammatory signals trigger local and systemic responses, resulting in a protective immune response against influenza virus (40, 41) and adaptive T and B cell responses (42).

The MF59 adjuvant has been shown, in mice and humans, to induce proinflammatory chemokines such as CXCL10 and cytokines [independently of type-1 interferon (IFN)] at the injection site, with recruitment of CD11b^+^ blood cells (43–45). These chemokines and cytokines promote more efficient antigen uptake by, and differentiation of, monocytes, macrophages and granulocytes, and differentiation of monocytes into immature dendritic cells (DCs) (46). MF59 also primes for enhanced processing and presentation of antigen for broader recognition of epitopes (47, 48).

There is extensive evidence, mainly from murine studies, to show that influenza HA-specific IgM can mediate protection from initial infection and re-infection (42, 49–54). In mice, innate B1 cells have a clearly defined role in systemic and local protection through spontaneous, steady state secretion of natural IgM antibodies (49, 51, 52). Murine models of influenza infection noted that B1a cell secretion of viral–specific IgM is enhanced locally, but not systemically, following infection (52, 53). While the systemic response was mediated by conventional, B2 cell-derived IgM (49, 51, 52). In human infants (aged 14–24 months), systemic serum IgM, IgM-plasma cells (PCs) and IgM-memory B cell responses, specific to vaccine H3N2 and H1N1 components, were observed 1 month after two doses of TIV or ATIV, although no difference was observed between the groups (29).

The role of IgM and innate B cells in human responses is not clearly understood since HAI titers are a measure of total Ab function and not just IgG; however, the contribution of IgM in influenza virus neutralization assays has been demonstrated (55, 56), so it could be proposed that IgM also has a role in hemagglutination.

### Memory B Cells (BMEM), Antibody Secreting Cells (ASCs), and Cross-Reactive Antibodies Are Enhanced by Adjuvant

Influenza infection induces a mucosal (nasopharygeal lamina propria) B cell, antibody and cellular response which is maintained over time (37). In mice it was shown that BMEM localized to the lung could provide protection from reinfection, while the bone marrow resident long-lived plasma cell (LLPC), spontaneously secreted antibody to provide immediate protection (57).

There seems to be some separation of the mucosal response from the systemic response (peripheral blood and tonsils) induced by intramuscular (i/m) immunization (37, 58). However, detection of PCs or ASC in peripheral blood may be the best marker of recent infection or response to immunization in naive subjects (59).

While there is very little information in the literature on BMEM and ASC responses in infants and very young children there are some age group comparisons. The ASC responses in adults versus children (aged 2–3 years) immunized with TIV were similar between primed children and adults, but in unprimed children only the IgM-ASC response was equivalent to adults, while the IgG and IgA-ASC response was significantly lower (60). IgG-ASC responses have also been detected in other age groups following either TIV [in children aged 6 months to 4 years (39)] or TIV versus MF59 ATIV immunization [in children aged 14–26 months (29), and in adults (61)]. However, even with adjuvant use in younger children, the day 7 peak in frequency of IgG-ASC in children is less than in adults, which may be related to maturity of the immune system and previous priming (61). The peak in ASC at day 7 postimmunization is almost always referring to IgG response; however, similar peaks in IgA, and to a lesser extent, IgM are also described (37).

Induction of IgG-BMEM has been observed in both primed and unprimed adults, although the magnitude of the response was enhanced in the presence of MF59 (38). In children aged 14–24 months both TIV and ATIV vaccines induced a greater frequency of IgM-BMEM than IgG-BMEM. However, the functional, HAI, responses were more robust and long lived following MF59-ATIV than after TIV (29).

Even in the absence of adjuvant, Influenza HA induces polyclonal stimulation of B cells and production of IgM antibodies, some of which are cross-reactive with different flu strains (62–64). *In vitro* studies have revealed HA stalk-specific antibodies that show different binding patterns, which indicates multiple conserved epitopes (65).

Specificity of ASC and antibody in response to TIV is more strain specific, with little cross-reactivity in comparison with controlled infection (H3N2) where ASC were reactive with a number of different strains (66). Repeated exposure *via* TIV immunization also limited induction of cross-reactive stem antibodies while response to the immunodominant head structure increased (67), suggesting that primary responses (in younger cohorts) induce stem antibodies while the recall response, mediated by BMEM and LLPCs (in older cohorts), is to the head structure (68).

Thus, the presence of preexisting HA-specific-BMEM may reduce (or focus) the breadth of subsequent Ab and ASC specificity and presence of high titers of serum HA-specific antibodies corresponds with a poorer ASC response (61, 69–71). It was suggested that cross-strain responses could be improved if strains included in the seasonal vaccines varied more frequently (70). However, in the presence of MF59-adjuvanted vaccines, more robust BMEM responses to clade mismatched H5 viruses (38) and A/strain group mismatched viruses (H5N1 vs. H7N9) were achieved than with TIV alone (69).

The cross-reactive antibodies undergo affinity maturation following immunization (H1N1-pdm09), which correlates with increased expression of activation-induced cytidine deaminase (72). MF59 and AS03 have been shown to enhance the production of cross-reactive (38, 73) and strain-specific antibodies compared with non-adjuvanted versions of the same influenza A/strains (46, 61, 65, 74–83). A similar effect was also seen with rintatolimod (a TLR-3 adjuvant), given intranasally with LAIV against H5 and H7 strains (84). However, this approach was not as successful for B/strains of the virus (79).

The role of somatic hypermutation, during memory B cell development, in broadening the cross-specificity of preexiting memory was described by Fu et al. (85) who demonstrated acquisition of H5 specificity following a single mutation of an H1/H3-specific germline VH sequence (IGVH3-30, Mab 3I14) directed against the HA stem.

The A/strain-specific cross-reactive antibodies have been identified following immunization in adults, the elderly (47, 75), and in children (31, 86). Generation of these cross-reactive antibodies is one of the main aims of new influenza vaccine development in order to help protect against future, related, pandemic strains (38, 61, 64, 71).

### Role of T Follicular Helper cells (Tfh) and Enhancement by Adjuvant

Kopf et al. (53) suggested a primary role for B1 cell-derived IgM may be to enhance CD4^+^ T cell priming at sites of infection. IgM-opsonized viral antigen may be captured by DCs that can prime T cell responses (53). IgM-Ag complexes may also flow back to draining lymph nodes (LNs), enhancing viral-specific CD4^+^ T cell-B cell interactions and subsequent germinal center formation (53).

Thus the enhanced recruitment of antigen presenting cells induced by MF59 to sites where these CD4^+^T cell-conventional B cell interactions are occurring may partly explain the enhanced IgG memory responses achieved by ATIV vaccines and that the innate and adaptive mechanisms are required to achieve protective responses.

The role of CD4^+^ T cells in supporting antibody responses against influenza HA has been accepted for many years (42). However, in recent years a subset of CD4^+^ T cells, known as T follicular helper (Tfh) cells have been strongly implicated to be involved in robust, long-lived antibody responses to influenza infection (87, 88) and immunization with TIV (89) and ATIV (90).

Tfh cells differentiate under certain conditions at the T-B cell border of the lymphoid follicles and require proinflammatory conditions (91). Activated B cells secrete IL-6 which induces Bcl6 expression and enhances IL-21 secretion by CD4^+^ T cells. IL-21 triggers differentiation of CD4^+^ T cells into Tfh cells which secrete IL-21, maintaining their function.

There is some redundancy and only mice deficient in both IL-6 and IL-21 fail to make Tfh responses (91). IL-7 has also been implicated in Tfh development (87).

Immunization of adults and children with TIV induced robust Tfh responses by day 7 postimmunization, but only in the presence of immune memory (89). In naive children there was limited Tfh response—as was observed in infant mice (92). The frequency of Tfh cells also correlated with rise in antibody HAI titers (89), and immunization using LAIV induced circulatory (c)Tfh responses that strongly correlated with increased antibody avidity and expansion of HA-specific Tfh clones (93, 94). The cTfh population was identified in the peripheral blood prior to immunization and characterized as CXCR5^+^PD1^+^ICOS^+^CD38^+^, with higher expression of CD27, CD25, CD28, CTLA4, PD1, Helios, and Ki67, but lower CD127 than total CD4^+^ T cells (94).

While influenza infection and administration of non-adjuvanted influenza vaccines induced robust Tfh responses in adults, addition of MF59 as an adjuvant significantly enhanced the response (95) with expansion of HA-specific Tfh (CD4^+^ICOS^+^, CD4^±^ICOS^+^CXCR5^+^IL-21^+^) by day 7 postimmunization that highly correlated with HAI titers 1 and 6 months later (95).

Previous infection or repeated immunization led to competition for virus-specific CD4^+^ T cells limiting naive Tfh expansion, but inducing expansion of preexisiting, clonal populations that subsequently resided in a memory population of ICOS-CD38-cTfh (94, 96).

Thus it could be proposed that cross-reactive, IgM antibodies, produced by innate B cells, trap antigen on DCs within the lymphoid follicles. This antigen activates B cells and is presented to CD4^+^ T cells, enhancing Tfh responses and B cell help, leading to robust, highly avid IgG antibody production. In naive infants, lacking preexisting pools of memory Tfh, the response to influenza would be predominated by IgM, but priming of a Tfh population would occur following infection or immunization. Thus subsequent immunization induces protective IgG responses. In adults, primed by infections, Tfh memory pools exist enabling strong IgG responses. The importance of adjuvants, such of MF59, therefore, may be to induce cross-reactive cellular subpopulations with each dose, helping to avoid narrowing of HA-specificities present within the memory populations, increasing likelihood of protection against future, related pandemic strains of influenza virus. Accordingly, MF59 was described in mice as mediating its adjuvanticity on influenza HA by promoting Tfh and thus Germinal Center responses in adult and early life—but not to fail inducing Tfh cells and thus humoral responses in neonatal mice (92).

### Innate Responses and Transcriptomes

A protective role for IFN-related genes during influenza infection has been demonstrated in mice (97) and in humans (98). A study of mice knocked-out for the IFN-inducible transmembrane protein (IFITM) demonstrated the importance of this gene in protection from severe influenza infection with enhanced pathogenesis and overproduction of proinflammatory cytokines (97). A human minor *IFITM* allele (SNP rs12252-C) was also associated with hospitalization in pandemic A(H1N1)pdm09 influenza patients (97). The SNP rs12252-C allele was further investigated and associated with influenza infection severity in a study of Chinese patients infected with severe pandemic A(H1N1)pdm09 (98).

Although these findings suggest a role of type I IFN in limiting viral replication, these cytokines might also play a role in modulating adaptive immune responses capable of eliciting better protection against reinfection. Indeed mouse studies have shown that adjuvants triggering innate immune responses *via* activation of innate immune receptors such as TLR4 and TLR7 are superior in inducing protective immunity when compared with vaccines unable to do so (99).

Squalene-based adjuvants such as MF59 and AS03 are also capable of triggering innate immune responses *via* a yet unknown mechanism.

A study (100) comparing the immune response induced by vaccines containing alum, TLR7 agonists and MF59 found that MF59 is far superior to alum alone in its capacity to promote immune cell infiltration to the injection site in the muscle, resulting in antigen uptake by neutrophils, monocytes, and myeloid and plasmacytoid DCs and migration exclusively to the vaccine-draining LNs. This resulted in priming of higher numbers of antigen-specific CD4^+^ T cells in the vaccine-draining LNs, increased T follicular helper cell differentiation and germinal center formation, and better antibody responses. Although this study failed to identify a type 1 IFN response in mice immunized with MF59, this adjuvant has been shown to induce increased IFN expression in infants (29). This innate response has been previously described in adults and is associated with stronger antibody responses (101). A similar observation was made in children aged 6 months to 14 years vaccinated with TIV or LAIV, where an association was found between upregulation of IFN genes at day 1 post-TIV and enhanced antibody responses, but only in children more than 5 years of age (102). In younger children (aged less than 5 years) IFN responses were not observed until day 7 post-LAIV (102).

A recent study (103) compared innate and adaptive immune responses in hepatitis B virus (HBV) naive individuals following receipt of a vaccine containing HBV surface antigen (HBsAg) adjuvanted with one of the following: AS01 [TLR4 ligand 3-*O*-desacyl-4′-monophosphoryl lipid A (MPL) and the purified saponin QS-21], AS03 (α-tocopherol and squalene in an oil-in-water emulsion), AS04 [MPL adsorbed on aluminum salt (AlPO_4_)], or Alum/Al(OH)3. Consistent with a role of innate responses in vaccine immunogenicity, the authors found that the adjuvanted vaccines capable of eliciting more pronounced antibody and CD4 T cell responses to HBsAg (AS01 and AS03), also induced an early mobilization of neutrophils and monocytes. Following vaccination with the AS01-adjuvanted vaccine, accumulation of cytokines, specifically IL-6, in serum was detectable as early as 3–6 h after vaccination. In addition, upregulation of IFN response genes was observed following the second dose of the AS01-adjuvanted vaccine but not following first or second dose of the other adjuvants. Notably, an increase in innate response and immunogenicity also correlated with more pronounced reactogenicity.

Recently, systems biological approaches have been used to define molecular signatures induced by vaccination in humans, and to understand their mechanisms of action. A systems-based approach was used to define the molecular signatures in response to vaccination with the live attenuated yellow fever vaccine (YF-17D), and to use such signatures to predict the immunogenicity of this vaccine (104). This offered proof of concept evidence of the utility of systems-based approaches in predicting vaccine immunity (104). In an independent study of the response to YF-17D, Sekaly and colleagues undertook a similar approach and obtained similar results (105). Subsequently, this approach has been extended to other vaccines such as the seasonal influenza vaccine (106–109), meningococcal vaccines (101) and shingles vaccines (110, 111), and in the infant population (29, 102). Importantly, recent studies have extended this approach to identifying signatures that predict vaccine-induced protection from disease (112, 113).

In addition, systems vaccinology approaches have been used to study responses to adjuvanted influenza vaccines. Both oil-in-water-based adjuvants, AS03 and MF59, induce specific transcriptional responses. These responses have been analyzed both in non-clinical mouse models and in clinical cohorts. In a non-clinical setting, upon injection of AS03 adjuvant, potent transcriptional responses have been observed both at the site of the injection and in the draining LNs as soon as 4 h postinjection (114). These changes affect a large number of chemotactic chemokines, believed to be involved in the recruitment of monocytes (CCL2, CCL3, CCL7), neutrophils (CXCL1, CXCL5, CXCL2, CSF3), eosinophils (CCL5), and DCs (CXCL9, CXCL10, CCL3, and CCL4). Of interest, while similar patterns of gene expression changes was observed in draining LNs, these changes tend to be more transient, with a peak at 4 h and diminishing signal at 24 h postinjection (114). It was also demonstrated that these responses were largely mediated by the α-tocopherol component of AS03, both in terms of the kinetics of the response, and the spectrum of chemokines being induced. *In vitro* studies identified monocytes and macrophages as the primary target cell type for α-tocopherol, and responsible for the production of chemokines in response to AS03 stimulation (114). In a clinical setting, a systems biology analysis of the effects of AS03 on responses to influenza vaccine has not yet been done in pediatric cohorts. However, in adults transcriptional responses in sorted cell populations were compared in cohorts receiving adjuvanted and non-adjuvanted H5N1 split-virion vaccine with high temporal resolution (115). This analysis demonstrated distinct gene expression patterns specific to distinct cell populations in peripheral blood, although different immune cell types responded at different time points. These responses were shown to correlate with cytokine production and antibody response (115). In another study, a large cohort of adult volunteers was followed longitudinally pre- and postadministration of A(H1N1) AS03 adjuvanted vaccine (Pandemrix^®^) (116). Early postvaccination the authors observed a transient decrease of expression of a large number of T cell-specific transcripts, accompanied by a strong upregulation of a large number of IFN response genes. Serum IFN gamma levels were accordingly elevated. Of special interest, age was a factor in gene expression patterns observed at day 1 postvaccination, with volunteers aged 35 or older demonstrating altered expression of several transcripts involved in early responses (116). While no pediatric subjects were included in this study, these results are relevant in light of the striking differences between responses to the same vaccine in infants and adults, discussed below.

Effects of MF59 on transcriptome have been extensively studies both in clinical setting (29) and mouse models (43, 44, 117), and reviewed by Olafsdottir et al. (118). MF59 induced strong localized transcriptional response, far exceeding in magnitude the response to CpG and Alum adjuvants (44), including the induction of a wide spectrum of cytokines and cytokine receptors, which included all (Alum), or nearly all (CpG), cytokines induced by other adjuvants. This potent transcriptional response was accompanied by a stronger recruitment of MHC class II and CD11b bearing cells to the site of injection (44). In a later study it was demonstrated that the observed effects on localized gene expression, cellular recruitment, antigen-specific humoral and T-cell responses, and antigen translocation to draining LN were all due to the combination of components of the adjuvant, as none of the individual components were able to elicit comparable responses (117). Further dissecting the functional transcriptional responses at the site of injection, Caproni et al. were able to demonstrate that the induction of proinflammatory genes, as well as genes relevant to transendothelial leukocyte migration correlated with the recruitment of CD11b^+^ cells to the site of injection, and antibody and cellular responses (43). Of interest, in a mouse model, MF59 induced very weak IFN type I response, and IFN signaling through its cognate receptor was not required for mounting potent humoral response (43).

**Figure 1:**
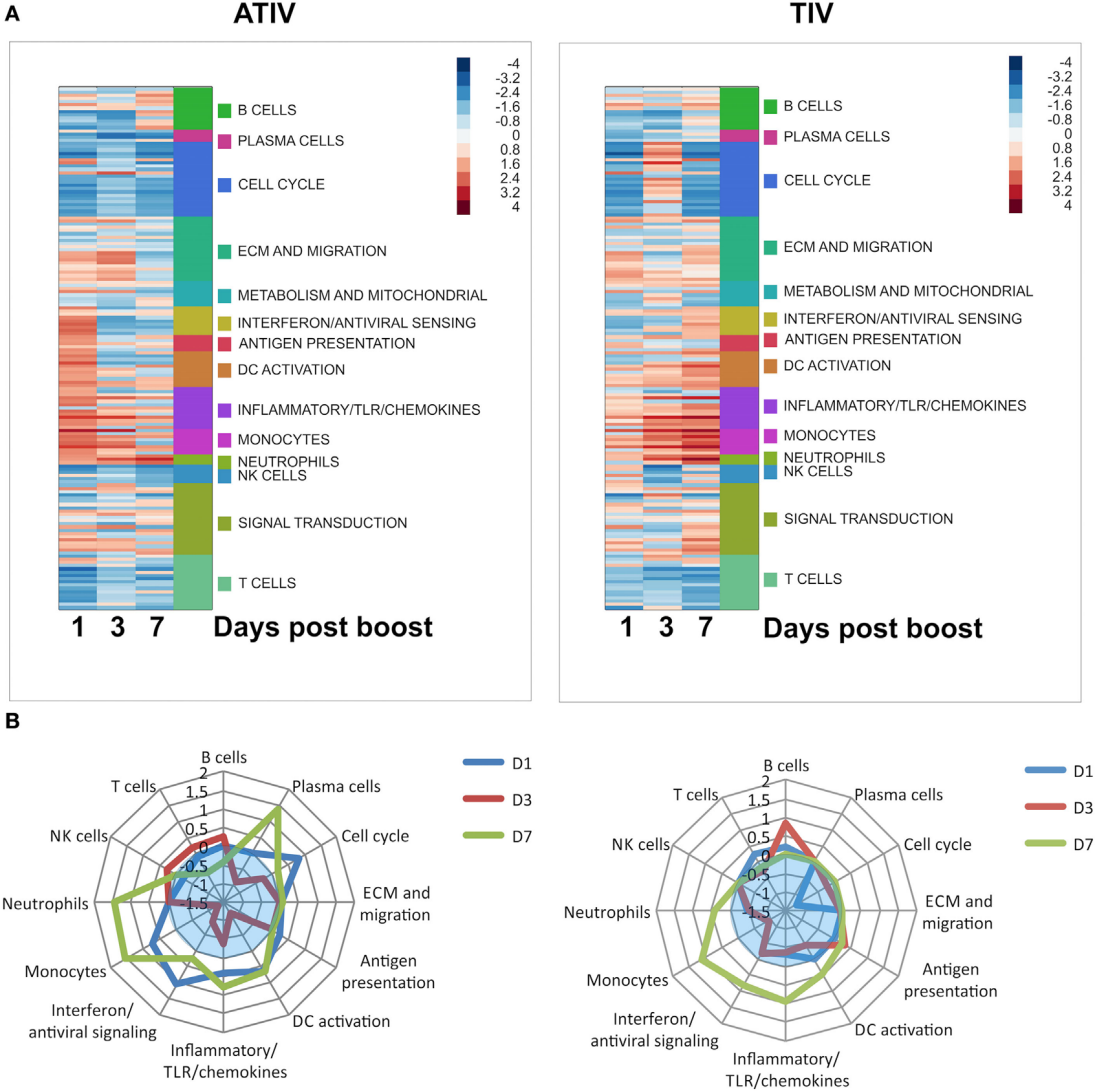
**(A)** Functional enrichment of transcriptional responses to adjuvanted trivalent influenza vaccine (ATIV) and trivalent influenza vaccine (TIV) vaccination. Enrichment scores generated by the GSEA analysis represented by color panel in right upper corner. Each row represents a module, which are grouped according to the high level notation group, as illustrated on the side bar. Positive enrichment indicates a combined upregulation of genes included in a module. **(B)** Correlates of immunogenicity (HAI titers) for the ATIV and TIV vaccines. GSEA was performed with genes ranked by correlation of expression to HAI titers. Distance from the center of the spider plot corresponds to the average enrichment score across all modules included in the high level annotation group. Only modules with enrichments significant by *p* < 0.05 are included; enrichments for insignificantly enriched modules are set to zero. Blue translucent zones indicate negative enrichment.

These results, however, were not recapitulated in a pediatric clinical study in which the effects of MF59 ATIV were compared with those of an unadjuvanted TIV (29). Indeed, in infants it was shown that MF59-adjuvanted TIV induces a strong and transient expression of a large number of IFN type I response genes, and that the induction of these genes at day 1 postboost vaccination tracked positively with HAI responses. Overall, MF59 ATIV induced a much stronger transcriptional response at day 1 postboost vaccination, although the magnitude of this response was much lower than in the adult cohorts investigated in a separate and independent study. These early responses were dominated by a large number of gene modules relevant to DC activation, antigen presentation, monocytes, IFN and antiviral response (Figure 1A). A notable feature of the responses to vaccination in infants is the high heterogeneity of responses. The unadjuvanted vaccine was able to induce gene expression patterns characteristic of innate immune responses in only a minority of subjects. In contrast, inclusion of MF59 adjuvant allowed the number of transcriptional responders to be pushed much higher, with only one subject still failing to mount an innate transcriptional response. Innate type transcriptional response early postvaccination is a correlate of immunogenicity in adults, and these correlates were recapitulated in the infants receiving MF59 ATIV. In contrast, the weak induction of an innate transcriptional response by unadjuvanted vaccines results in the lack of such correlates at day 1 postvaccination. In fact, it takes 7 days for the vaccines in an unadjuvanted arm to develop the spectrum of transcriptional correlates of immunogenicity similar to correlates that are evident in adjuvanted arm as early as day 1 postvaccination (Figure 1B). Even then, the correlations between the expression of innate immunity gene modules and HAI titers are weaker and encompass fewer modules than in the adjuvanted arm. Together, these data suggest that unadjuvanted vaccines induce weak and delayed innate transcriptional response, resulting in lower HAI titers, while the inclusion of MF59 adjuvant allows the development of a stronger and more uniform innate response, and a spectrum of transcriptional correlates of antibody responses resembling correlates observed in adults. An intriguing observation made in this study was an inverse relationship between the correlates of antibody responses observed at day 3 postvaccination in infants and those in adults. Further studies are currently underway to directly compare the effects of MF59 ATIV in adults and infants and to shed more light on the molecular mechanism of action of MF59-adjuvanted influenza vaccine in pediatric population.

## Efficacy and Effectiveness

### MF59

The efficacy of MF59 ATIV against RT-PCR-confirmed influenza infection was assessed in a randomized controlled phase III study in Germany and Finland, including 4,707 children aged 6–72 months across two consecutive influenza seasons. MF59 ATIV was compared with a conventional TIV and control (meningococcal C conjugate vaccine) (119). The absolute vaccine efficacy against all influenza virus strains for the MF59 ATIV was 86% (95% CI, 74–93) compared with 43% (95% CI, 15–61) for the non-adjuvanted TIV, with a relative efficacy of 75% (95% CI, 55–87) (119). These results are supported by the superior immunogenicity of MF59 ATIV seen in the same study. Given the limited use of TIV and LAIV in children less than 2 years of age, TIV secondary to immunogenicity and LAIV secondary to safety concerns, it is particularly important to highlight that the efficacy of MF59 ATIV in children aged 6–24 months against matched strains was 75% (95% CI, 20–92) compared with 2% for TIV. It should be noted that the overall efficacy in this study predominantly reflects protection against H3N2 (94 of the 110 culture-confirmed influenza cases were H3N2). Based on these promising study results and others, an application for marketing authorization with the European Medicines Agency (EMA) was submitted to the Committee for Medicinal Products for Human Use (CHMP). During the application process concerns were raised by the CHMP regarding flaws in good clinical practice (GCP) in the clinical trial described above. This application was withdrawn in 2012 following the initial assessment by the CHMP due to ongoing unresolved issues regarding the concerns with compliance with GCP (120).

Currently, there are no postlicensure data available to assess the effectiveness of MF59 ATIV in children. However, this information should be available in the future given the recent licensure of MF59 ATIV in Canada for children aged 6 months to 2 years.

There are limited data on the effectiveness of the monovalent A/H1N1 MF59 adjuvanted vaccine in children during the 2009 H1N1 pandemic. The MF59 adjuvanted pandemic vaccine Focetria^®^ was one of several pandemic vaccines available and millions of doses were administered to children mainly in Europe and Latin America. The effectiveness against ILI and laboratory confirmed A(H1N1)pdm09 infection was estimated in the Netherlands; however, the children included were only those with an underlying medical condition indicating their need for vaccination. In the total cohort there was a crude vaccine effectiveness (VE) against ILI of 17.3% (95% CI, −8.5–36.9%). For children aged between 5 and 19 years the adjusted VE against ILI was 51% (95% CI, −50–84%) and VE of children 4 years or less was not able to estimate due to the small number of children included (121). One report from Spain did not show significant VE in children aged 1–17 years against medically attended ILI (VE: 12%, 95% CI, −142–68%) (122). Lastly, a recently published systematic review of 2009 pandemic influenza A(H1N1) vaccines did not find any studies fulfilling inclusion criteria that included children who had received an MF59-adjuvanted vaccine (123).

The monovalent, cell culture-derived inactivated subunit vaccine adjuvanted with MF59 (Celtura^®^) gained local regulatory approval in four countries in Europe and Latin America (Germany, Switzerland, Chile, and Peru). However, there have not been any published studies reporting the effectiveness of this vaccine in children.

Overall, despite some promising results in this published literature, further efficacy and effectiveness data are required for MF59 adjuvanted vaccines to strengthen the argument for licensure in young children.

### AS03-Adjuvanted Pandemic Vaccines

The VE and efficacy in children of the AS03-adjuvanted A(H1N1) vaccine has been assessed in many studies, including three systematic reviews (123–125). The recently published systematic review by Lansbury et al. (123) reported pooled VE against laboratory-confirmed influenza from four studies with a total of 932 children (126–129). Pooled VE was estimated at 88% (95% CI, 69–95%, *p* < 0.0001) for adjuvanted H1N1 vaccines compared with 45% (95% CI, −13–73%, *p* = 0.83) for non-adjuvanted vaccines. This difference was statistically significant and the result did not differ if the studies included were limited to only those which measured VE from 14 days after vaccination. Pooled VE against hospitalization due to laboratory-confirmed influenza A(H1N1) illness was estimated at 86% (95% CI, 67–94%, *p* < 0.00001) using results from two studies (130, 131). The majority of studies assessing effectiveness were done in Europe and Canada, as described below.

One multinational RCT reported a vaccine efficacy against RT-PCR confirmed influenza of 76.8% (95% CI, 18.5–93.4%) in children aged 6 months to less than 10 years of age receiving an AS03-adjuvanted vaccine (Arepanrix^®^, GSK) compared with non-adjuvanted vaccine during 2010–2011 influenza season (132).

#### Europe

The EMA recommended the pandemic vaccine (Pandemrix^®^ in most countries) should, in the first instance, be provided to “risk groups,” which included children less than 2 years of age, followed by “target groups” (which included children of all ages over 6 months). Target groups were offered vaccination in a staggered fashion throughout Europe, with children often being included in the early stages to help reduce transmission and provide indirect protection. For example, in the United Kingdom children 6 months and older who were in a clinical risk group were eligible for a two 0.25 ml dose course of Pandemrix^®^ vaccine initially. This was changed in December 2009 due to the increasing numbers of hospital admissions in children, and all healthy children aged 6 months to 5 years were eligible for one 0.25 ml dose (133).

Effectiveness of AS03-adjuvanted H1N1 vaccine (Pandemrix^®^) against RT-PCR positive A(H1N1) influenza infection was extensively evaluated in Stockholm County, Sweden. In Sweden at the time of the study two 0.25 ml doses were recommended for children aged 6 months and older, with a vaccination coverage of 52% for children aged 6 months to 2 years receiving at least one vaccine dose and 70% in children aged 3–18 years. The estimated VE for children aged 6 months to 12 years during the peak weeks of the 2009–2010 season was 89–92%, with most children having only received one dose of the vaccine (134). VE against hospitalization (used as a surrogate indicator for severe disease) in children aged 6 months to 17 years due to influenza in the same population, adjusted for comorbid conditions, was 91% (130). Örtqvist et al. followed this cohort in a long term effectiveness study in the subsequent influenza seasons (135). During the 2010–2011 influenza season the adjusted VE for those vaccinated with Pandemrix^®^ in 2009 was estimated at 91.7%; however, during the 2012–2013 season, there was no evidence of ongoing protection from previous vaccination. There was almost no H1N1 virus circulating in the 2011–2012 season therefore VE was unable to be estimated. Very few children received the seasonal influenza vaccine in seasons following the 2009 pandemic, likely due parents’ concerns regarding safety, therefore the long-term effectiveness reported here is thought to truly reflect the 2009 vaccination program rather than vaccination in subsequent seasons.

Similarly impressive results were observed in an English case control study which estimated a VE against laboratory-confirmed influenza in children aged less than 10 years of 77% (CI, 11–94) and 100% (95% CI, 80–100) in those aged 10–24 years (126). In another case control study across England and Scotland including over 2000 children aged less than 15 years no vaccine failures occurred, therefore the VE estimate in children less than 15 years of age was 100% (95% CI, 74–100) (136).

The comparison of VE between these studies is difficult due to varying methods used to estimate VE, the broad range of age groups, differing number of doses administered and a variety of approaches to collect confirmed influenza infection cases. Two methods to estimate the VE of the pandemic vaccine were compared in a German population, one a test-negative case-control method using virologic surveillance data and the other an innovative case-series methodology using nationally reported laboratory-confirmed influenza case data (128). In children less than 14 years of age the estimate of VE using both methods were similar, with the first estimating a VE of 79% (95% CI, 35–93, *p* = 0.007) and the second 87% (95% CI, 78–92, *p* < 0.001).

#### Canada

In Canada, children aged 3–10 years were recommended to receive a single 0.25 ml dose of an AS03-adjuvanted A(H1N1) vaccine (Arepanrix^®^), and children 6 months to less than 3 years of age to receive two doses. In the Canadian province Manitoba, the VE against laboratory-confirmed influenza cases was estimated at 97% (95% CI, 72–100) in very young children (aged 6–35 months) compared with no protection provided by the seasonal TIV (127). Although statistically significant results for this same age group were not found in another community-based study due to small sample size, an even higher VE against laboratory-confirmed influenza of 100% (95% CI, 79.5–100) was reported in children aged 3 years to less than 10 years (129).

Varying results have been reported regarding VE against pneumonia and hospitalization in Canada. In Quebec, the effectiveness of a single pediatric vaccine dose in preventing hospitalization due to influenza in children aged 6 months to 9 years was 85% (95% CI, 61–94) (131), whereas the VE against hospitalization was considerably less, 58% (30–75%), for children less than 5 years of age in another study (137).

## Safety

### MF59-Adjuvanted Influenza Vaccines

There are extensive data regarding the safety of MF59-adjuvanted influenza vaccines which have demonstrated an acceptable safety profile in young children (26, 27, 30, 32, 33, 119, 138–147). Most adverse reactions are mild-to-moderate and transient in nature and serious adverse reactions are rare. A systematic review and meta-analysis has provided an overview of safety for both seasonal and pandemic MF59-adjuvanted influenza vaccines in children (144). The analysis reported no increase in serious adverse events (SAEs) compared with control vaccines. The rate of SAEs in the adjuvanted group was 0.0–10.4% with a pooled relative risk of 0.74 (95% CI, 0.57–0.97) (144). The relative risk for the most common solicited adverse events including redness and pain at the injection site and systemic reactions such as fever, irritability and loss appetite were significantly higher for MF59-adjuvanted vaccines compared with control vaccines. The rates of solicited adverse reactions included 1.0–59.0% for pain (<1% for grade 3 pain) and 4.0–19.0% for fever (144). There were similar rates of unsolicited adverse event reporting between children who received adjuvanted compared with non-adjuvanted vaccines.

An integrated analysis evaluated the safety of MF59-adjuvanted vaccines (predominantly the seasonal trivalent or tetravalent vaccine) in children 6 months to 18 years of age (139). The analysis included five clinical trials—four trials with a seasonal MF59-adjuvanted vaccine and one trial with the prepandemic H5N1 vaccine. A total of 1,181 children received an MF59-adjuvanted vaccine compared with 545 children who received a non-adjuvanted vaccine. There was an increased incidence in solicited local and systemic reactions compared with non-adjuvanted vaccines; however, these were mostly mild and transient, resolving by day 3 postvaccination. Across all ages 55% experienced local reactions and 48% systemic reactions after the first dose in the MF59-adjuvanted groups, and 43% and 34%, respectively, in the non-adjuvanted group. These were slightly lower in both groups following the second vaccination. There was no difference in the rate of SAEs.

Following this analysis, safety data have been published in large immunogenicity and efficacy studies. The study by Vesikari et al. reported minimal difference in local reactions between adjuvanted and non-adjuvanted influenza vaccines aged 6–36 months, and fever was reported in 15.3 versus 13.3%, respectively (119). Similar results were demonstrated in a large phase III, randomized, multicenter study which included 3,125 children who received ATIV (143).

Studies focusing on the pandemic H1N1 and prepandemic H5N1 vaccines have also shown MF59 to have an acceptable safety profile in children (31, 148–156). Transient mild pain or tenderness and erythema were the most commonly reported local reactions and fatigue and myalgia the most common systemic reactions. Few, if any, children reported severe reactions including fever >40°C.

Theoretical concerns have been raised that MF59 vaccination may induce antibodies to squalene. Squalene is a naturally occurring product in the body and antibodies to the squalene component of the vaccine would therefore pose a risk of autoimmune disease in a vaccine recipient. Subsequent studies have demonstrated that vaccination with MF59 adjuvant did not induce antisqualene antibodies nor enhance preexisting antisqualene antibody levels (157).

Despite the association between the AS03-adjuvanted pandemic vaccine Pandemrix^®^ and narcolepsy (described below), there has been no evidence to date of any increased risk of narcolepsy associated with MF59-adjuvanted vaccines in children or adults (158); however, postlicensure surveillance in children will be important to continue monitoring for this as the frequency reported for Pandemrix^®^ was too low to detect in clinical trials.

### AS03-Adjuvanted Influenza Vaccines

Prior to the licensing of the pandemic AS03-adjuvanted influenza vaccines, evidence on the safety of the AS03 adjuvant was available from clinical trials, which demonstrated an acceptable reactogenicity profile (159–161). Following the rapid licensure of Pandemrix^®^ in Europe and Arepanrix^®^ in Canada, passive and active surveillance programs were initiated. During the 2009–2010 influenza season 31 million doses of Pandemrix^®^ were distributed throughout Europe, and 12 million doses of Arepanrix^®^ mainly in Canada and Latin America (162) with the collection of safety data *via* these national surveillance programs. With limited safety data available prior to its distribution, it was not until postlicensure surveillance revealed concerns regarding Pandemrix^®^ in children.

Clinical trials have demonstrated acceptable rates of solicited local and systemic adverse events, albeit higher than non-adjuvanted vaccines, following vaccination with the AS03-adjuvanted pandemic vaccines (132, 163–170). A recent systematic review included four clinical trials enrolling children who received an AS03-adjuvanted influenza vaccine. There were significantly increased rates of local adverse reactions including pain and swelling, although mostly mild and transient, after receiving the AS03-adjuvanted vaccine compared with non-adjuvanted control vaccines (144). Pooled data from these studies showed local pain as the most frequent adverse event following AS03-adjuvanted vaccines in children, experienced by 31.7–84.6% of children, with rates of grade 3 pain between 4.3 and 12.4%. The rate of fever following vaccination was 11.0–23.8% and there was no significantly increased risk of developing an unsolicited adverse event (RR 1.0, 95% CI, 0.97–1.04) or convulsion (RR 1.14, 95% CI, 0.42–3.14) compared with non-adjuvanted vaccines. Moreover, there was no increased risk of SAEs, with 0.0–8.0% of children experiencing an SAE. There is evidence in children that the second vaccine dose, given 21–28 days after the first, results in higher rates of local and/or systemic reactions compared with the first dose, although this is not consistent across all studies (132, 163, 166). Immunocompromised children have experienced similar rates of adverse events compared with immunocompetent children (171, 172).

The EMA announced on August 27, 2010, that a safety review had been initiated following concerns raised in Sweden with a case series of six adolescents diagnosed with narcolepsy within 2 months of vaccination with Pandemrix^®^ (173, 174). An investigation by the European Centre for Disease Prevention and Control (ECDC) and Vaccine Adverse Event Surveillance and Communication Consortium (VAESCO) was then undertaken in late 2010 (175). Following the initial report, formal studies assessing an association between Pandemrix^®^ and narcolepsy have been undertaken in Finland, Sweden, France, Ireland, United Kingdom, and Norway which have confirmed an increased incidence of narcolepsy in young vaccine recipients (176–182). In Finnish children aged 4–19 years there was a rate ratio of 12.7 (95% CI, 6.1–30.8), with a vaccine-attributable risk of 1:16,000 (95% CI, 1:13,000–1:21,000) of developing narcolepsy following receipt of Pandemrix^®^ (176). The EMA Eudravigilance database had received almost 1,400 reports of narcolepsy in Pandemrix^®^-recipients by 2015 (183).

Narcolepsy is a rare sleep condition with onset often in adolescence which is characterized by excessive daytime sleepiness, episodes of unintended sleep and cataplexy. It is thought to be due to immune-mediated destruction of neurons which results in deficiency in hypocretin production in the hypothalamus, although no specific antibodies involved in this process have been identified. The majority of individuals with narcolepsy and cataplexy express the HLADQB1*0602 allele, and infections including influenza A and *Streptococcus pyogenes* have been implicated in triggering narcolepsy in susceptible individuals (184).

The biological plausibility linking Pandemrix^®^ and narcolepsy has been explored although the exact mechanism is yet to be identified. The AS03 adjuvant itself and specific components of AS03 (e.g., α-tocopherol) not present in other adjuvants were suggested to be responsible; however, the lack of association between other AS03-containing vaccines (e.g., Arepanrix^®^) and narcolepsy may refute this theory (162, 176, 185). Molecular mimicry has been proposed as a possible mechanism behind the association, with one study reporting a similarity between a peptide on the influenza nucleoprotein A and an extracellular domain of the hypocretin receptor 2 (186). This study was subsequently retracted due to inability to replicate results but, despite this, did not adequately explain why narcolepsy would not be associated with other H1N1 vaccines. Further studies investigating the presence of neuronal antibodies have not identified narcolepsy-specific antibodies in the sera or CSF of vaccinated children with narcolepsy (187).

Arepanrix^®^, the AS03-adjuvanted pandemic vaccine used in Canada, has not been associated with such a significant risk of narcolepsy, with only one extra case of narcolepsy per million doses received (183, 188). This is despite both Pandemrix^®^ and Arepanrix^®^ vaccines containing similar amounts of HA and AS03. The different method of production of the H1N1 antigen between the two vaccines, resulting in antigenic differences, has been suggested to result in enhanced levels of IgG-antibodies that may be implicated in the association of Pandemrix^®^ with narcolepsy (189). However, in a separate study sera and CSF samples from 13 vaccinated patients with narcolepsy were compared with 44 vaccinated patients without narcolepsy, revealing no increase in narcolepsy-specific autoantibodies (187).

## Conclusion

Conventional influenza vaccines have suboptimal immunogenicity in young children and adjuvanted influenza vaccines offer a superior alternative. MF59 and AS03 have proven to be immunogenic in young children, provide cross protection against mismatched influenza virus strains and allow for antigen sparing which is important in the setting of pandemics where the global demand is high. Despite these positive results, the association between AS03 and narcolepsy has resulted in the future use of the current AS03 formulation in children limited. MF59 ATIV is efficacious in children leading to its licensure in Canada in children; however, further studies investigating the effectiveness of MF59 seasonal vaccines would potentially improve the likelihood of licensure in young children in other countries and more widespread use of this vaccine in children.

Looking to the future, the recent advancements in understanding the mechanism of adjuvants through elucidating the innate and adaptive immune response and relating these with gene expression profiles will allow both the improvement of current adjuvants and development of novel adjuvants. The progress with some newer adjuvanted influenza vaccines is promising. Adjuvants based on toll-like receptor agonists including TLR4 and TLR9 agonists used in pandemic and “universal” influenza vaccines, respectively, have shown excellent results in phase I trials. A variety of bacteria-derived adjuvants (e.g., flagellin, *Escherichia coli* heat-labile toxin patch, meningococcal outer membrane protein) which take advantage of the ability of bacterial components to activate the innate immune system have been incorporated in seasonal trivalent influenza vaccines, with some moving to phase III clinical trials. New technologies have allowed the development of these adjuvants among a myriad of others (e.g., liposomes, virus–like particles, saponins, viral vectors, and newer oil-in-water emulsions); however, many remain in the experimental phase in animals and there is a lack of robust human data for the majority of these currently. Finally, a “universal” influenza vaccine which provides protection against all influenza virus strains, regardless of antigen drift or shift, with long-lasting protection remains an ultimate goal in the development of improved influenza vaccines. The combination of novel antigen formulations and adjuvants underlies many candidate vaccines currently in development. A number of these vaccines have entered clinical trials in recent years with the most advanced (recombinant M2e fused with flagellin, VAX102) reaching phase II trials. These vaccines face the challenge of providing an equivalent, if not better, immunogenic response than current seasonal influenza vaccines and ensuring an acceptable safety profile. Given it has been 20 years since the licensure of an MF59-containing vaccine and licensure of an influenza vaccine adjuvanted with MF59 for children has only occurred recently, it is likely to be some time before adjuvanted vaccines are widely used in the pediatric population.

The aim continues to be provision of the best possible protection of children from influenza while minimizing reactogenicity. That no vaccine against influenza is yet licensed for the most vulnerable, less than 6-month-old term or preterm-born infants is a challenge that may not remain unaddressed.

## Author Contributions

AW, DK, GN, and EC wrote the initial drafts for the manuscript which was then revised and contributed to by BP, C-AS, and AP.

## Conflict of Interest Statement

C-AS has received numerous educational or research grants, including from vaccine manufacturers, although none related to this work. AP has previously conducted clinical studies on behalf of Oxford University that were sponsored by vaccine manufacturers with grants from Okairos and Pfizer closing since January 2015. His department received unrestricted educational grants from Novartis/GSK/Astra Zeneca in 2015, Pfizer/GSK/Astra Zeneca in July 2016, and Gilead/MSD/GSK/Astra Zeneca in June 2017 to support a 3-day course on Infection and Immunity in Children. He is Chair of UK Dept. Health’s (DH) Joint Committee on Vaccination and Immunization (JCVI) and chair of the scientific advisory group on vaccines for the European Medicines Agency and is a member of the WHO’s SAGE. The views expressed in this manuscript do not necessarily represent the views of DH, JCVI or WHO. All other authors declare that the research was conducted in the absence of any commercial or financial relationships that could be construed as a potential conflict of interest.
